# Supplementary material for: Effects of Repeated Cisplatin and Monosodium Glutamate on Visceral Sensitivity in Rats
Source: Cells. 2024 Dec 30;14(1):26. doi: 10.3390/cells14010026 (PMC11719532; doi:10.3390/cells14010026)
Supplement: Supplementary file 1 [file cells-14-00026-s001.zip › cells-3245446-supplementary.pdf]

cells

MDPI

Supplementary

Effects of Repeated Cisplatin and Monosodium Glutamate on Visceral Sensitivity in Rats

Yolanda López-Tofiño [1,2,3], Laura López-Gómez [1,2], Marta Martín-Ruíz [1], Jose Antonio Uranga [1,2], Kulmira Nurgali [4,5,6], Gema Vera [1,2,7,*] and Raquel Abalo [1,2,3,7,8,*]

1 Department of Basic Health Sciences, University Rey Juan Carlos (URJC), 28922 Alcorcón, Spain; yolanda.lopez@urjc.es (Y.L.-T.); laura.lopez.gomez@urjc.es (L.L.-G.); martamruiz5@gmail.com (M.M.-R.); jose.uranga@urjc.es (J.A.U.)
2 High Performance Research Group in Physiopathology and Pharmacology of the Digestive System (NeuGut), University Rey Juan Carlos (URJC), 28922 Alcorcón, Spain
3 Working Group of Basic Sciences on Cannabinoids of the Spanish Pain Society, 28046 Madrid, Spain
4 Institute for Health and Sport, Victoria University, Melbourne, VIC 3021, Australia; kulmira.nurgali@vu.edu.au
5 Department of Medicine Western Health, The University of Melbourne, Melbourne, VIC 3010, Australia
6 Regenerative Medicine and Stem Cell Program, Australian Institute for Musculoskeletal Science (AIMSS), Melbourne, VIC 3021, Australia
7 Associated R+D+i Unit to the Institute of Medicinal Chemistry (IQM), Scientific Research Superior Council (CSIC), 28006 Madrid, Spain
8 Working Group of Basic Sciences on Pain and Analgesia of the Spanish Pain Society, 28046 Madrid, Spain
* Correspondence: gema.vera@urjc.es (G.V.); raquel.abalo@urjc.es (R.A.); Tel.: +34-914889086 (G.V.); +34-914888854 (R.A.)

Supplementary figure

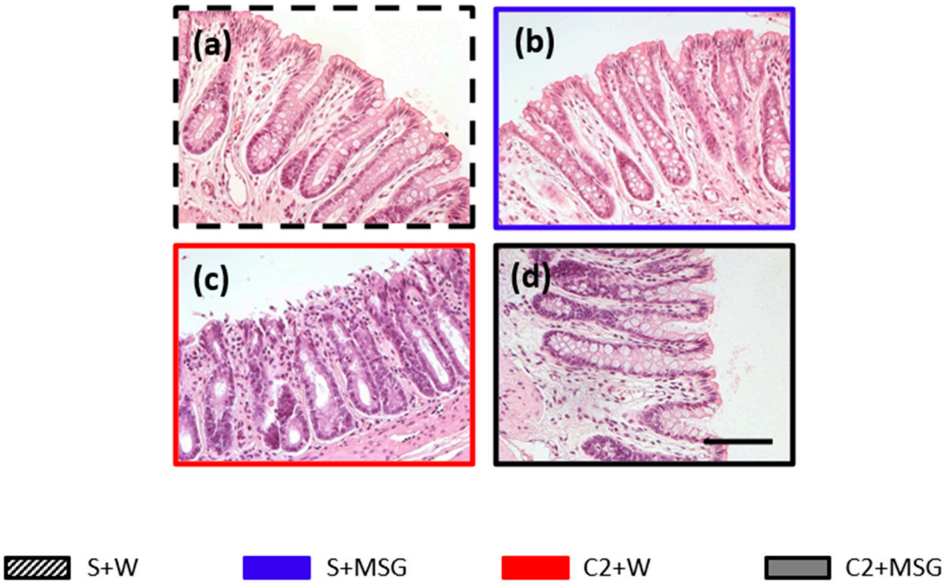

Figure S1. Representative images of the effect of cisplatin and monosodium glutamate (MSG) in the distal colon. The rats were intraperitoneally administered with saline (2.5 mL/kg, S) or cisplatin (2 mg/kg, C2) for 5 consecutive weeks (weeks 1–5) and exposed or not to MSG (4 g/L) in drinking water (W) from week 0 to 1 week after treatment (week 6). At the end of the experiment (week 6), distal colon samples were embedded in paraffin, sectioned, stained with hematoxilin-eosin. (a) saline + water; (b) saline + MSG; (c) cisplatin + water; (d) cisplatin + MSG. Bar: 100 µm.
